# Diet and Survival in Black Women With Epithelial Ovarian Cancer

**DOI:** 10.1001/jamanetworkopen.2024.40279

**Published:** 2024-10-18

**Authors:** Tsion A. Armidie, Elisa V. Bandera, Courtney E. Johnson, Lauren C. Peres, Kristin Haller, Paul Terry, Maxwell Akonde, Edward S. Peters, Michele L. Cote, Theresa A. Hastert, Lindsay J. Collin, Michael Epstein, Jeffrey Marks, Melissa Bondy, Andrew B. Lawson, Anthony J. Alberg, Joellen M. Schildkraut, Bo Qin

**Affiliations:** 1 Department of Epidemiology, Rollins School of Public Health, Emory University, Atlanta, Georgia; 2 Cancer Epidemiology and Health Outcomes, Rutgers Cancer Institute, New Brunswick, New Jersey; 3 Department of Cancer Epidemiology, H. Lee Moffitt Cancer Center and Research Institute, Tampa, Florida; 4 Department of Medicine, University of Tennessee Medical Center-Knoxville; 5 Department of Epidemiology and Biostatistics, University of South Carolina Arnold School of Public Health, Columbia; 6 Department of Epidemiology, College of Public Health, University of Nebraska Medical Center, Omaha; 7 Indiana University Melvin and Bren Simon Comprehensive Cancer Center, Indiana University, Indianapolis; 8 Department of Oncology, Wayne State University School of Medicine, Detroit, Michigan; 9 Department of Population Health Sciences, Huntsman Cancer Institute, University of Utah, Salt Lake City; 10 Department of Surgery, Duke University School of Medicine, Durham, North Carolina; 11 Department of Epidemiology and Population Health, Stanford University School of Medicine, Stanford, California; 12 Department of Public Health Sciences, Medical University of South Carolina, Charleston; 13 Usher Institute, Centre for Population Health Sciences, Edinburgh Medical School, University of Edinburgh, Edinburgh, United Kingdom

## Abstract

**Importance:**

Ovarian cancer survival among Black women is the lowest across all racial and ethnic groups. Poor dietary quality also disproportionately affects Black populations, but its association with ovarian cancer survival in this population remains largely unknown.

**Objective:**

To examine associations between dietary patterns and survival among Black women diagnosed with epithelial ovarian cancer (EOC).

**Design, Setting, and Participants:**

This prospective cohort study was conducted among self-identified Black women aged 20 to 79 years newly diagnosed with histologically confirmed EOC in the African American Cancer Epidemiology Study (AACES) between December 2010 and December 2015, with follow-up until October 2022. AACES is a population-based study of ovarian cancer risk and survival among Black women in 11 US regions. Data were analyzed from March 2023 to June 2024.

**Exposures:**

Dietary patterns were assessed by the Healthy Eating Index-2020 (HEI-2020) and Alternative Healthy Eating Index-2010 (AHEI-2010), with scores calculated based on dietary intake in the year prior to diagnosis and collected via the validated Block 2005 Food Frequency Questionnaire. Higher scores indicate better dietary quality.

**Main outcomes and measures:**

Hazard ratios (HRs) and 95% CIs were estimated from multivariable Cox models for the association between adherence to dietary recommendations and overall mortality among all participants and those with high-grade serous ovarian cancer (HGSOC).

**Results:**

Among 483 Black women with EOC (mean [SD] age, 58.1 [10.5] years), 310 deaths were recorded during a median (IQR) follow-up of 4.3 (2.0-8.2) years. No association of dietary patterns with mortality was found among women with EOC overall. However, among 325 women with HGSOC, better adherence to HEI-2020 was associated with decreased mortality in later quartiles compared with the first quartile (HR, 0.63; 95% CI, 0.44-0.92 for quartile 2; HR, 0.67; 95% CI, 0.46-0.97 for quartile 3; HR, 0.63; 95% CI, 0.44-0.91 for quartile 4 ). Similar results were observed with AHEI-2010 among women with HGSOC for the second (HR, 0.62; 95% CI, 0.43-0.89) and fourth (HR, 0.67; 95% CI, 0.45-0.98) quartiles compared with quartile 1.

**Conclusions and relevance:**

In this study, women with moderate and high prediagnosis dietary quality had significantly lower mortality rates from HGSOC compared with women with the lowest prediagnosis dietary quality. These findings suggest that even moderate adherence to dietary guidelines prior to diagnosis may be associated with improved survival among Black women with HGSOC, the most lethal form of ovarian cancer.

## Key Points

**Question:**

Is adherence to dietary recommendations associated with survival among Black women after an epithelial ovarian cancer diagnosis?

**Findings:**

In this cohort study of 483 Black women diagnosed with epithelial ovarian cancer, there was no significant difference in overall mortality according to diet for the full sample. However, prediagnosis moderate and high adherence to the 2020 to 2025 Dietary Guidelines for Americans were associated with decreased mortality for the high-grade serous histotype compared with the lowest adherence.

**Meaning:**

These findings suggest that for Black women with the lowest prediagnosis adherence to dietary guidelines, modest improvements in dietary quality may be associated with improved survival for the most lethal epithelial ovarian cancer histotype.

## Teaser

This cohort study investigates the association of adherence to dietary guidelines with survival among Black women with epithelial ovarian cancer.

## Introduction

Ovarian cancer is the sixth leading cause of cancer deaths among women in the US.^1,2^ Approximately 90% of ovarian cancers are epithelial ovarian cancer (EOC), with most being high-grade serous ovarian cancer (HGSOC), the most lethal histotype.^2^ The 5-year relative survival rate is 51% overall, and it is 42% among African American or Black women (hereafter, *Black women*), which is lower compared with other racial and ethnic groups.^2^ Therefore, it is imperative to identify strategies, including modifiable lifestyle factors, to improve survival of ovarian cancer, especially for Black women.

A healthy dietary pattern is recommended for cancer prevention and survivorship,^3,4^ but there is limited research evaluating the role of dietary patterns specifically in ovarian cancer survival. Dietary pattern analysis captures the complex combinations of foods and nutrients and their overall outcomes. In our previous study from the African American Cancer Epidemiology Study (AACES),^5^ we evaluated dietary patterns using 2 index-based scores, the Healthy Eating Index-2010 (HEI-2010) and Alternative Healthy Eating Index-2010 (AHEI-2010). We found that adherence to an overall healthy dietary pattern was associated with reduced risk of EOC among postmenopausal Black women. The HEI-2020 and AHEI-2010 are the latest adaptations of these indices, which align with the 2020 to 2025 Dietary Guidelines for Americans^6^ and the Healthy Eating Pyramid,^7^ respectively. Existing evidence on dietary pattern indices and ovarian cancer survival has yielded inconsistent findings and has primarily included populations of predominantly White women,^8,9,10,11,12^ despite poor dietary quality disproportionately affecting Black populations.^13^ Therefore, we examined the association of adherence to dietary recommendations as assessed by the HEI-2020 and AHEI-2010 with survival among a cohort of Black women with EOC. To our knowledge, this has not been investigated in a population-based study of Black women with ovarian cancer.

## Methods

### Study Population and Design

This prospective cohort study used data from AACES, a multisite, population-based study of ovarian cancer risk and survival in Black and African American women diagnosed between December 2010 and December 2015. Details regarding AACES have been described elsewhere.^14,15^ In brief, rapid case ascertainment from state cancer registries; Surveillance, Epidemiology, and End Results (SEER) registries; or hospitals was used to identify cases. Patients were included if they were newly diagnosed and histologically confirmed to have invasive EOC, self-reported as African American or Black females, aged 20 to 79 years, residents of 1 of 11 US states (New Jersey, Illinois, Michigan, Ohio, Georgia, North Carolina, South Carolina, Alabama, Tennessee, Louisiana, or Texas), and English speaking. At baseline, information on sociodemographics, lifestyle and reproductive factors, and medical history were collected by telephone survey. Information on diet was collected through the validated and self-administered Block 2005 Food Frequency Questionnaire (FFQ), which was mailed to participants.^16,17,18,19^ Clinical characteristics, including tumor characteristics, first-line treatment regimen, debulking status, residual disease, and cancer antigen 125 levels, were abstracted from medical records. Of 592 eligible patients, 489 women completed the FFQ (median [IQR] time since diagnosis, 5.8 [3.5-9.7] months). We excluded 6 participants who reported extreme total energy intake (>2 × the IQR of log energy intake^5^) or had missing histotype, giving a final sample size of 483 individuals. Informed written or verbal consent was obtained from all participants in AACES, which covers this study.^15^ The Institutional Review Boards of Duke University Medical Center, the University of Virginia, and participating sites approved the study protocol. This study followed the Strengthening the Reporting of Observational Studies in Epidemiology (STROBE) reporting guideline for cohort studies.

### Dietary Exposure Assessment

The FFQ gathers information on the consumption of 110 foods and beverages in the year preceding diagnosis. The HEI-2020 has 13 components separated into adequacy (ie, foods to eat in greater quantities for health: total fruits, whole fruits, total vegetables, greens and beans, whole grains, dairy, total protein foods, seafood and plant proteins, and unsaturated to saturated fatty acids ratio) and moderation (ie, foods to limit for health: refined grains, sodium, added sugars, and saturated fats) categories. HEI-2020 scores range from 0 to 100, with a higher score indicating greater adherence to the guideline.^6^ The AHEI-2010 has 11 food and nutrient components:^7,20^ vegetables, fruits, whole grains, nuts and legumes, long-chain (n-3) fats, polyunsaturated fatty acids, sugar-sweetened beverages and fruit juices, red and processed meat, trans fat, sodium, and alcohol.^7^ AHEI-2010 scores range from 0 to 110, and a higher score indicates better dietary quality.^7^

### Outcome Ascertainment

The study outcome was overall survival. Vital status and date of death were ascertained annually through multiple sources, including cancer registries, the National Death Index, the LexisNexis database, and patient contact. To avoid immortal time bias, survival time was calculated as the number of days from interview date to the date of death or last contact (October 2022, except for Detroit, Michigan, which was March 2022).

### Statistical Analysis

Participant characteristics were summarized across quartiles of HEI-2020 and AHEI-2010 scores, and χ^2^ or *t* tests were used for comparison. Kaplan-Meier survival curves and log-rank tests were used to compare survival by quartile of each dietary pattern score. Adjusted hazard ratios (HRs) and 95% CIs for the association of dietary patterns with mortality were estimated by Cox proportional hazard regression models. The proportional hazards assumption was assessed through the Schoenfeld test, and no violation was found. The model was adjusted for the following covariates, which were selected based on prior evidence and a directed acyclic graph: age, education, household income level, meeting physical activity recommendations,^21^ smoking status, study site, and histotype. We also considered other covariates, including health insurance, dietary supplement use, total energy intake, BMI (body mass index; calculated as weight in kilograms divided by height in meters squared), diabetes status, tumor stage, and debulking status, but found that they were not confounders and that adjusting for them did not materially change estimates. To evaluate a linear trend, the median value of each quartile was modeled as a continuous variable in Cox models.

We repeated all analyses among women with HGSOC, the most common and lethal histotype. Additionally, we investigated whether the association between dietary pattern and survival was modified by age at diagnosis, prediagnosis obesity status, diabetes status, tumor stage, or debulking status, with *P* values for multiplicative interaction estimated using the likelihood ratio test.

To examine how each food or nutrient component contributed to the association between overall dietary pattern scores and overall survival, we estimated the association between a 1-point increase in each component and survival, adjusting for the overall score without that component. We also estimated the association between dietary pattern score and survival by removing 1 component at a time. Statistical significance was defined as a 2-sided *P* < .05. Statistical analyses were performed using Stata statistical software version 17.0 (StataCorp), and graphics were generated using SAS statistical software version 9.4 (SAS Institute). Data were analyzed from March 2023 to June 2024.

## Results

Our study included 483 Black women with EOC (mean [SD] age at ovarian cancer diagnosis, 58.1 [10.5] years). During a median (IQR) follow-up of 4.3 (2.0-8.2 years) since interview, 310 deaths were recorded. Participants had a mean (SD) score of 67.9 (9.5) for HEI-2020 and 53.1 (10.2) for AHEI-2010 (eTable 1 in Supplement 1). By HEI-2020 score, the mean amount consumed by women in our study was close to the recommended amount for greens and beans, total protein foods, seafood, and plant proteins, as well as limited amounts of refined grains. However, the consumption of whole grains and dairy was much lower than recommended, and sodium intake was high. By AHEI-2010 score, the mean amount consumed by women in our study was close to the recommended amount for polyunsaturated fatty acids but much lower than the recommended amounts for fruits, whole grains, and n-3 fats, and there was excessive consumption of trans fats and sugar-sweetened beverages and fruit juices.

Among all women in this study, 325 individuals (67.3%) had HGSOC (Table 1). Participants with lower education levels, current smokers, and those who did not use dietary supplements were more likely to have lower scores for HEI-2020 and AHEI-2010. Additionally, women with lower HEI-2020 scores tended to be younger and had a higher total energy intake, while those with lower AHEI-2010 scores were less likely to meet physical activity guidelines.

**Table 1.** Study Participant Characteristics

Characteristic	Participants, No. (%)^a^						
	All (N = 483)	HEI-2020			AHEI-2010		
		Q1 (n = 121)^b^	Q4 (n = 120)^b^	*P* value	Q1 (n = 121)^b^	Q4 (n = 120)^b^	*P* value
Age, mean (SD), y	58.1 (10.5)	56.1 (11.0)	58.8 (10.7)	.02	58.1 (10.8)	57.9 (9.92)	.89
Site							
Southwest	122 (25.3)	34 (28.1)	26 (21.7)	.71	32 (26.4)	23 (19.2)	.28
Southeast	256 (53.0)	65 (53.7)	64 (53.3)		64 (52.9)	66 (55.0)	
North	105 (21.7)	22 (18.2)	30 (25.0)		25 (20.7)	31 (25.8)	
Annual household income, $							
<25 000	197 (43.1)	52 (46.0)	40 (36.0)	.29	56 (50.9)	39 (32.5)	.06
≥25 000	260 (56.9)	61 (54.0)	71 (64.0)		54 (49.1)	78 (65.0)	
Unknown	26	8	9	NA	11	3	NA
Education							
High school or less	238 (49.3)	65 (53.7)	44 (36.7)	.01	74 (61.2)	40 (33.3)	<.001
Some college	93 (19.3)	26 (21.5)	32 (26.7)		21 (17.4)	35 (29.2)	
College graduate	152 (31.5)	30 (24.8)	44 (36.7)		26 (21.5)	45 (37.5)	
Marital status							
Single	109 (22.6)	28 (23.1)	25 (20.8)	>.99	26 (21.5)	24 (20.0)	.92
Married	166 (34.4)	40 (33.1)	45 (37.5)		37 (30.6)	46 (38.3)	
Divorced	138 (28.6)	35 (28.9)	31 (25.8)		40 (33.1)	34 (28.3)	
Widowed	70 (14.5)	18 (14.9)	19 (15.8)		18 (14.9)	16 (13.3)	
Insurance status							
None	46 (10.0)	15 (13.2)	11 (9.7)	.99	12 (9.9)	14 (11.9)	.59
Any Medicaid	94 (20.4)	25 (21.9)	21 (18.4)		30 (24.8)	17 (14.4)	
Medicare only	103 (22.3)	22 (19.3)	24 (21.1)		23 (19.0)	23 (19.5)	
Private insurance and Medicare	24 (5.2)	5 (4.4)	8 (7.0)		6 (5.0)	9 (7.6)	
Private insurance only or with other insurance	168 (36.4)	40 (35.1)	44 (38.6)		33 (27.3)	46 (40.0)	
Other	27 (5.8)	7 (6.1)	6 (5.3)		7 (5.8)	9 (7.6)	
Unknown	21	7	6	NA	10	2	NA
Menopausal status							
Premenopausal	122 (25.3)	35 (28.9)	33 (27.5)	.03	28 (23.1)	30 (25.0)	.31
Postmenopausal	361 (74.7)	86 (71.1)	87 (72.5)		93 (76.9)	90 (75.0)	
BMI^c^							
<25	71 (14.8)	19 (15.7)	18 (15.1)	.15	17 (14.1)	19 (15.8)	.41
25-29.99	129 (26.8)	27 (22.3)	39 (32.8)		28 (23.1)	35 (29.2)	
≥30-34.99	135 (28.1)	37 (30.6)	31 (26.1)		34 (28.1)	28 (23.3)	
≥35	146 (30.4)	38 (31.4)	31 (26.1)		42 (34.7)	38 (31.7)	
Unknown	2	0	1	NA	0	0	NA
Total energy intake, mean (SD), kcal/d	1720 (1080)	1900 (1130)	1490 (816)	.01	1720 (787)	1770 (1170)	.89
Smoking status							
Current smoker	81 (16.8)	25 (20.7)	7 (5.8)	.01	30 (24.8)	9 (7.5)	.03
Former smoker	132 (27.3)	28 (23.1)	32 (26.7)		31 (25.6)	35 (29.2)	
Never smoker	270 (55.9)	68 (56.2)	81 (67.5)		60 (49.6)	76 (63.3)	
Meets physical activity guidelines^d^							
Yes	120 (25.9)	25 (22.1)	34 (29.8)	.62	21 (18.9)	32 (27.1)	.05
No	343 (74.1)	88 (77.9)	80 (70.2)		90 (81.1)	86 (72.9)	
Unknown	20	8	6	NA	10	2	NA
Supplement use before diagnosis							
No	176 (36.4)	56 (46.3)	33 (27.5)	.03	63 (52.1)	28 (23.3)	<.001
Yes	307 (63.6)	65 (53.7)	87 (72.5)		58 (47.9)	92 (76.7)	
Charlson Comorbidity Index, No. conditions							
0	179 (37.1)	41 (33.9)	53 (44.2)	.27	37 (30.6)	53 (44.2)	.22
1	120 (24.8)	36 (29.8)	25 (20.8)		37 (30.6)	20 (16.7)	
≥2	184 (38.1)	44 (36.4)	42 (35.0)		47 (38.8)	47 (39.2)	
Talc use							
Ever	306 (63.4)	80 (66.1)	78 (65.0)	.72	79 (65.3)	71 (59.2)	.39
Never	177 (36.6)	41 (33.9)	42 (35.0)		42 (34.7)	49 (40.8)	
Aspirin use							
Yes	78 (17.0)	20 (17.7)	12 (10.7)	.18	19 (17.3)	16 (13.8)	.52
No	380 (83.0)	93 (82.3)	100 (89.3)		91 (82.7)	100 (86.2)	
Unknown	25	8	8	NA	11	4	NA
Nonaspirin NSAID use							
Yes	102 (22.3)	27 (23.9)	19 (17.0)	.36	28 (25.5)	27 (23.3)	.53
No	356 (77.7)	86 (76.1)	93 (83.0)		82 (74.6)	89 (76.7)	
Unknown	25	8	8	NA	11	4	NA
Histology							
HGSOC	325 (67.3)	75 (62.0)	86 (71.7)	.44	82 (67.8)	76 (63.3)	.72
Other	158 (32.7)	46 (38.0)	34 (28.3)		39 (32.2)	44 (36.7)	
Stage							
I and II	150 (33.0)	35 (30.2)	41 (36.6)	.69	32 (27.6)	45 (40.5)	.21
III and IV	305 (67.0)	81 (69.8)	71 (63.4)		84 (72.4)	66 (59.5)	
Unknown	28	5	8	NA	5	9	NA
Debulking status/CA-125^e^							
Optimal	340 (70.4)	80 (66.1)	94 (78.3)	.07	80 (66.1)	93 (77.5)	.16
Suboptimal	143 (29.6)	41 (33.9)	26 (21.7)		41 (33.9)	27 (22.5)	
Vital status with cause of death							
Alive	173 (35.8)	41 (33.9)	45 (37.5)	.53	38 (31.4)	50 (41.7)	.43
Deceased							
Ovarian cancer	201 (41.6)	54 (44.6)	47 (39.2)		55 (45.5)	45 (37.5)	
Other cause	45 (9.3)	10 (8.3)	9 (7.5)		8 (6.6)	11 (9.2)	
Unknown cause	64 (13.3)	16 (13.2)	19 (15.8)		20 (16.5)	14 (11.7)	

Abbreviations: AHEI-2010, Alternative Healthy Eating Index-2010; BMI, body mass index (calculated as weight in kilograms divided by height in meters squared); CA-125, cancer antigen 125; HEI-2020, Healthy Eating Index-2020; HGSOC, high-grade serous ovarian cancer; NA, not applicable; NSAID, nonsteroidal anti-inflammatory drug; Q, quartile.

^a^ Participants with unknown values for variables were excluded from analyses comparing groups for that variable.

^b^ Q1 and Q4 indicate low and high adherence to dietary guidelines, respectively. HEI-2020 score ranges were 61.03 or less for Q1, 61.04 to 67.93 for Q2, 67.94 to 74.20 for Q3, and 74.21 or more for Q4. AHEI-2010 score ranges were 45.33 or less for Q1, 45.34 to 52.47 for Q2, 52.48 to 59.68 for Q3, and 59.69 or more for Q4.

^c^ BMI was based on self-reported weight and height 1 year before diagnosis.

^d^ Physical activity in the year before diagnosis meets guidelines of 75 minutes or more of strenuous physical activity per week, 150 minutes or more of moderate physical activity, or an equivalent combination.

^e^ Missing values for debulking status were imputed in the African American Cancer Epidemiology Study.

**Figure 1.**
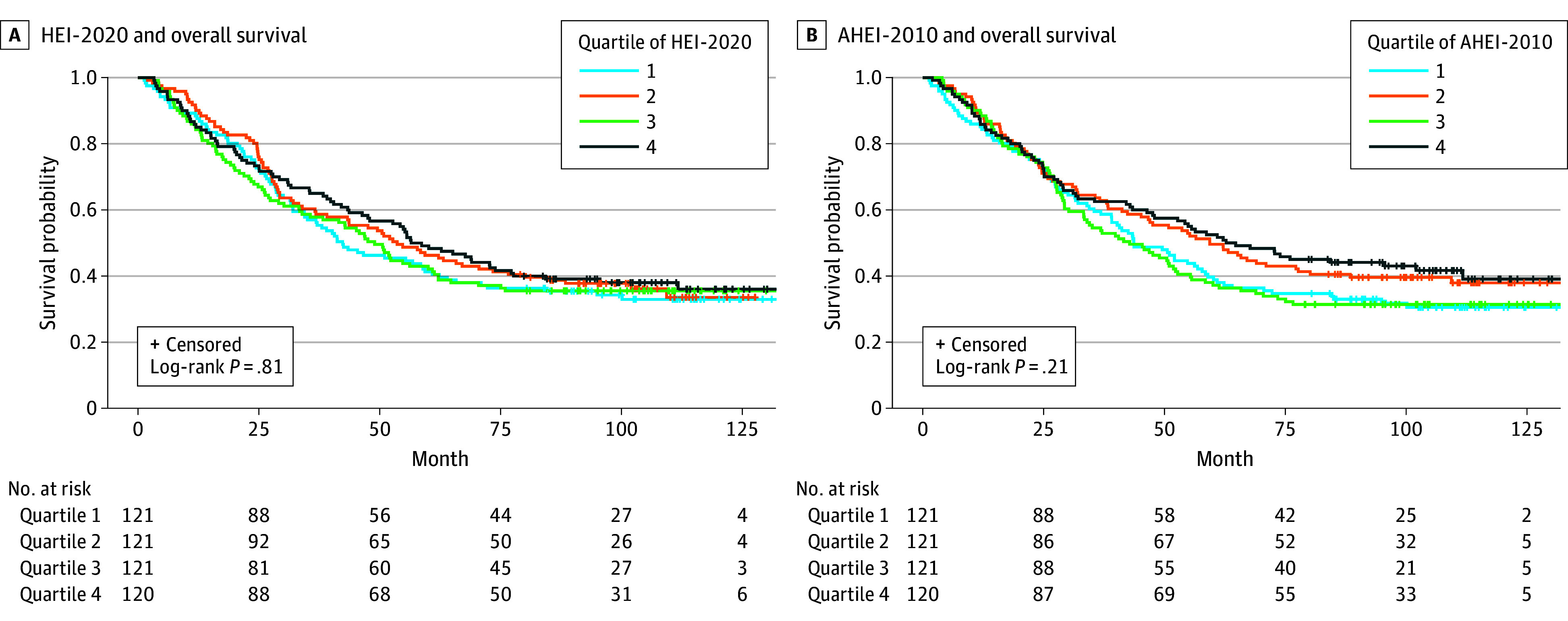
Dietary Patterns and Overall Survival in Epithelial Ovarian Cancer Unadjusted Kaplan-Meier curves show no difference in overall survival comparing quartiles of Healthy Eating Index-2020 (HEI-2020) scores (A) and Alternative Healthy Eating Index-2010 (AHEI-2010) scores (B).

Kaplan-Meier survival curves were similar across quartiles of HEI-2020 or AHEI 2010 score (Figure 1). In the multivariable model, there was no significant difference in overall mortality according to index score among women with EOC. However, women who were most adherent to dietary guidelines by quartile (ie, in quartile 4) had decreased mortality (HEI-2020: HR, 0.78; 95% CI, 0.56-1.08; AHEI-2010: HR, 0.83; 95% CI, 0.89-1.16) compared with those with the lowest adherence (ie, in quartile 1) (Table 2).

**Table 2.** Associations Between Dietary Pattern Scores and Overall Survival

Dietary pattern score	All participants (n = 483)				HGSOC (n = 325)			
	HEI-2020^a^		AHEI-2010^a^		HEI-2020^a^		AHEI-2010^a^	
	Deaths/participants, No.	HR (95% CI)^b^	Deaths/participants, No.	HR (95% CI)^b^	Deaths/participants, No.	HR (95% CI)^b^	Deaths/participants, No.	HR (95% CI)^b^
Quartile^c^								
1	80/121	1 [Reference]	83/121	1 [Reference]	63/75	1 [Reference]	68/82	1 [Reference]
2	77/121	0.82 (0.59-1.12)	74/121	0.80 (0.58-1.10)	56/83	0.63 (0.44-0.92)	53/82	0.62 (0.43-0.89)
3	78/121	0.87 (0.63-1.20)	83/121	1.02 (0.75-1.40)	56/81	0.67 (0.46-0.97)	64/85	0.92 (0.65-1.31)
4	75/120	0.78 (0.56-1.08)	70/120	0.83 (0.89-1.16)	58/86	0.63 (0.44-0.91)	48/76	0.67 (0.45-0.98)
*P* value for trend	NA	.19	NA	.52	NA	.03	NA	.17
Per 10-point increase	NA	0.93 (0.82-1.05)	NA	0.95 (0.85-1.07)	NA	0.86 (0.75-1.00)	NA	0.89 (0.77-1.01)

Abbreviations: AHEI-2010, Alternative Healthy Eating Index-2010; HGSOC, high-grade serous ovarian cancer; HEI-2020, Healthy Eating Index-2020; HR, hazard ratio; NA, not applicable.

^a^ Score ranges were 0 to 100 for HEI-2020 and 0 to 110 for AHEI-2010.

^b^ The Cox proportional hazard model was adjusted for age (years), education (≤high school graduate, some college, ≥college graduate), annual household income (<$25 000, ≥$25 000, or unknown), physical activity in the year before diagnosis (yes or no), smoking status (never, current, or former smoker), study site (Southwest, Southeast, or North), and histotype (HGSOC or other). In the analysis among patients with HGSOC, histotype was not adjusted.

^c^ Quartile 1 and Q4 indicate low and high adherence to dietary guidelines, respectively.

**Figure 2.**
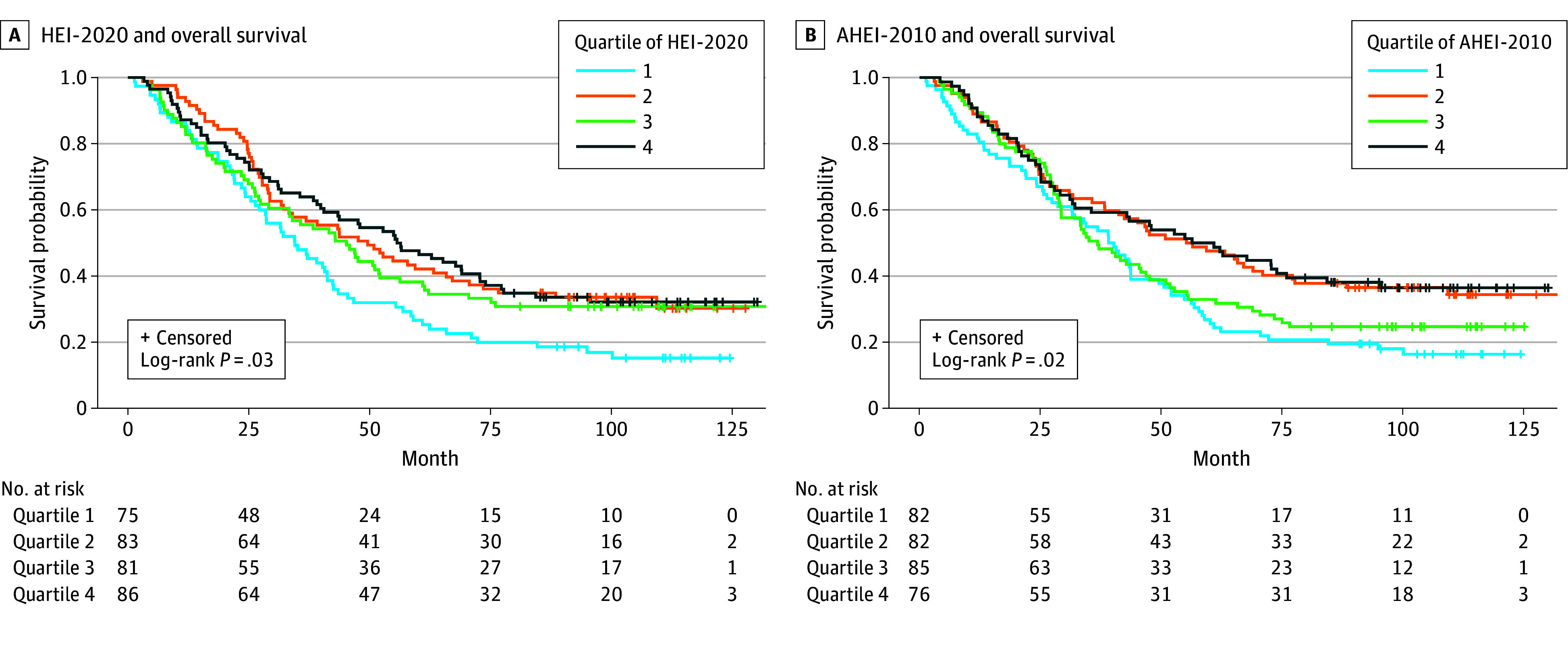
Dietary Patterns and Overall Survival in High-Grade Serous Ovarian Cancer Unadjusted Kaplan-Meier curves show differences in overall survival among those with high-grade serous ovarian cancer comparing quartiles of Healthy Eating Index-2020 (HEI-2020) scores (A) and Alternative Healthy Eating Index-2010 (AHEI-2010) scores (B).

Among women with HGSOC, those with higher prediagnosis dietary pattern scores had significantly better overall survival, as shown by Kaplan-Meier curves (Figure 2). In multivariable-adjusted models, women in the second quartile of HEI-2020 scores had a decreased mortality (HR, 0.63; 95% CI, 0.44-0.92) compared with those in the lowest quartile. The decreased mortality was also evident in higher quartiles of HEI-2020 compared with quartile 1 (quartile 3: HR, 0.67; 95% CI, 0.46-0.97; quartile 4: HR, 0.63; 95% CI, 0.44-0.91; *P* for trend = .03) (Table 2). For every 10-point increase in HEI-2020 score, there was a decrease in mortality (HR, 0.86; 95% CI, 0.75-1.00). Comparable lower mortality was found with AHEI-2010 scores among women with HGSOC who were in the second (HR, 0.62; 95% CI, 0.43-0.89) and fourth (HR_, _0.67; 95% CI, 0.45-0.98) quartiles compared with those in the first quartile.

We explored food and nutrient components in HEI-2020 and AHEI-2010 to identify components contributing to the association with mortality observed in women with HGSOC. We found consistent evidence comparing models evaluating each individual dietary component with models evaluating dietary scores without each individual component (eTable 2 in Supplement 1). We did not identify a single dietary component contributing most of the association observed in the 2 scores. However, compared with other dietary components in HEI-2020 and AHEI-2010, better adherence to low saturated fat intake by HEI-2020 score (HR per 1-point increase, 0.95; 95% CI, 0.90-1.00) and low red or processed meat consumption by AHEI-2010 score (HR per 1-point increase, 0.94; 95% CI, 0.90-0.98) were associated with greater decreases in mortality risk among women diagnosed with HGSOC (eTable 2 in Supplement 1).

We did not observe any interaction by tumor stage (eTable 3 in Supplement 1) or debulking status (eTable 4 in Supplement 1) in associations between dietary patterns and mortality. Additionally, there were no interactions by diabetes status, age, or prediagnostic obesity status. However, our results suggested that the association between HEI-2020 score by quartile and mortality was limited to women with a BMI less than 30 (HR for quartile 4 vs quartile 1, 0.57; 95% CI, 0.33-0.98; *P* for trend = .10) (Table 3), whereas there were no associations among women with a BMI of 30 or greater.

**Table 3.** Associations Between Dietary Pattern Scores and Overall Survival by Obesity Status

Dietary pattern score	HEI-2020^a^				AHEI-2010^a^			
	BMI ≥30 (n = 281)^b^		BMI <30 (n = 200)		BMI ≥30 (n = 281)^b^		BMI <30 (n = 200)	
	Deaths/participants, No.	HR (95% CI)^c^	Deaths/participants, No.	HR (95% CI)^c^	Deaths/participants, No.	HR (95% CI)^c^	Deaths/participants, No.	HR (95% CI)^c^
Quartile^d^								
1	47/75	1 [Reference]	33/46	1 [Reference]	52/76	1 [Reference]	31/45	1 [Reference]
2	50/73	1.04 (0.69-1.58)	27/48	0.54 (0.32-0.90)	45/78	0.72 (0.48-1.08)	28/42	0.89 (0.52-1.52)
3	45/71	0.97 (0.63-1.49)	32/49	0.68 (0.41-1.15)	46/61	1.10 (0.73-1.67)	36/59	0.89 (0.54-1.48)
4	42/62	0.93 (0.60-1.45)	32/57	0.57 (0.33-0.98)	41/66	0.89 (0.58-1.37)	29/54	0.73 (0.41-1.30)
*P* value for trend	NA	.74	NA	.10	NA	>.99	NA	.32
Per 10-point increase	NA	0.98 (0.83-1.15)	NA	0.84 (0.69-1.04)	NA	0.97 (0.83-1.13)	NA	0.93 (0.77-1.12)

Abbreviations: AHEI-2010, Alternative Healthy Eating Index-2010; BMI, body mass index (calculated as weight in kilograms divided by height in meters squared); HEI-2020, Healthy Eating Index-2020; HR, hazard ratio; NA, not applicable.

^a^ Score ranges were 0 to 100 for HEI-2020 and 0 to 110 for AHEI-2010. The *P* for interaction was .33 for HEI-2020 and .67 for AHEI-2010.

^b^ With obesity.

^c^ The Cox proportional hazard model was adjusted for age (years), education (≤high school graduate, some college, ≥college graduate), annual household income (<$25 000, ≥$25 000, or unknown), physical activity in the year before diagnosis (yes or no), smoking status (never, current, or former smoker), study site (Southwest, Southeast, or North), and histotype (HGSOC or other).

^d^ Quartile 1 and quartile 4 indicate low and high adherence to dietary guidelines, respectively.

## Discussion

In this population-based cohort study of Black women with EOC, dietary quality was not associated with survival overall. However, among women with HGSOC, the most common and lethal type of ovarian cancer, better prediagnostic dietary quality as evaluated by HEI-2020 (adherence to the Dietary Guidelines for Americans 2020-2025) and AHEI-2010 was associated with a decrease in mortality. The decreased mortality was observed from the second quartile of the dietary quality scores compared with the lowest quartile, suggesting that even modest improvements in dietary quality among women with the lowest adherence to dietary guidelines may be associated with increased survival rates in HGSOC. To our knowledge, this is the first study to evaluate the association of adherence to dietary guidelines with ovarian cancer survival among Black women, who have a disproportionate prevalence of lower dietary quality and worse ovarian cancer survival compared with other racial and ethnic groups.^1,13^

A few studies have examined dietary patterns and ovarian cancer survival, most of which were among White women, with inconsistent results.^8,9,10,11,12,22,23^ Using a previous iteration of HEI (HEI-2010), the Ovarian Cancer Prognosis and Lifestyle (OPAL) study among Australian women found no association of HEI score with ovarian cancer survival.^8^ In contrast, the Women’s Health Initiative (WHI) and National Institutes of Health-American Association of Retired Persons (NIH-AARP) Diet and Health study, both including predominantly White women with ovarian cancer, reported significant decreases in mortality (27% and 25%, respectively) comparing the highest level of HEI-2005 adherence with the lowest.^10,12^ The OPAL study and the Nurses’ Health Study (NHS) examined dietary quality using AHEI-2010, and both found no associations,^8,9^ which was similar to our results among all women with EOC. Among these 4 studies, the NHS also reported null findings across histotypes, whereas the NIH-AARP study observed associations in the nonserous epithelial histotype.^9,12^ Additionally, 3 studies^9,22,23^ have evaluated the association of dietary inflammatory potential with ovarian cancer survival using Empirical Dietary Inflammatory Pattern and Dietary Inflammatory Index scores. A proinflammatory diet was associated with worse survival among patients with non-HGSOC in the NHS, which included mostly White women, and among patients with HGSOC in the AACES, which included only Black women.^9,23^ The correlation between Dietary Inflammatory Index and HEI-2020 or AHEI-2010 was moderate in our study (*r* = −0.6).

We observed better survival for higher quartiles of prediagnosis dietary quality compared with the lowest quartile among Black women with HGSOC, while the prior evidence among White women was mixed. This could be due to several reasons. First, according to 2017 to 2018 National Health and Nutrition Examination Survey data, dietary quality as measured by HEI-2015 was notably lower among Black populations compared with White populations (HEI-2015 is identical to HEI-2020 because the dietary guidelines remain unchanged).^13^ Therefore, our study may have a reference group whose dietary quality was lower than that in studies among White women with ovarian cancer. Differences by racial and ethnic group in food preparation preferences^24^ and pathophysiology (eg, insulin sensitivity at a specific adiposity measure differs between Black and White individuals^25^) may have also contributed to our findings. Dietary pattern scores in our study were similar to those reported among Black women in other cohorts. For example, 50% of women in our study had an HEI-2020 score below 67.9. Comparatively, close to half (45%) of Black women in the Multiethnic Cohort Study had a score of 70.2 or less.^26^ In the WHI, 51% of Black women had a score of 65.4 or less,^27^ and in the Sister Study, 55% of women had a score below 72.8.^28^

Our findings suggest that prediagnosis dietary patterns (ie, the combination of foods and nutrients) are more important than individual components for ovarian cancer survival as shown by comparing results of dietary patterns with individual components. In ancillary analyses, no specific components of HEI-2020 or AHEI-2010 contributed most of the overall association observed in women with HGSOC. Among our study participants, the prediagnosis consumption of whole grains, dairy, fruit, and n-3 fats was much lower than the recommendations by HEI-2020 or AHEI-2010, whereas consumption of sodium, sugar-sweetened beverages, and fruit juices greatly exceeded recommended levels. The potential benefits associated with high dietary quality were observed from the second quartile and higher, suggesting that even moderate adherence to dietary guidelines may be associated with improved ovarian cancer survival rates for women with HGSOC. However, it should be acknowledged that consuming a high-quality diet is a privilege not afforded to all. Higher dietary quality has been associated with higher socioeconomic status^29^ and access to healthy foods.^3,30^ The associations we observed were independent of individual socioeconomic status; however, strategies to improve dietary quality among women with the lowest levels of dietary guideline adherence need to target social determinants of health factors, such as affordability and access to healthy foods.

Mechanisms through which a high-quality prediagnosis dietary pattern may be associated with improved survival after an HGSOC diagnosis remain unclear, but several pathways are potentially involved. Our study and others showed that HEI-2020 and AHEI-2010 are anti-inflammatory dietary patterns.^31,32,33^ There is evidence supporting the role of inflammation in the development of HGSOC and suggesting that chronic inflammation and a proinflammatory tumor microenvironment may play essential roles in the progression, metastasis, and chemoresistance of EOC, including HGSOC.^34^ The tumor’s immune landscape may be shaped by responses to inflammation-related factors,^35^ and significant racial differences have been observed in overall immune responses and in gene expression of key cytokines, with cells from individuals of African descent generally showing higher activation.^36^ Furthermore, higher diet quality contributes to better nutritional status and may be associated with preserved muscle mass during treatment^37^ and thereby potentially reduced treatment-related toxic effects, increased adherence, and, in turn, better survival after ovarian cancer diagnosis.^38,39^ We also observed that the association between HEI-2020 adherence and overall survival was present only among women who were not obese. Patients with cancer and obesity are more likely to already experience metabolic dysregulation, including elevated systemic inflammation,^40^ which may diminish survival benefits associated with a high-quality diet. The WHI study similarly observed that the association between HEI-2005 and overall survival after ovarian cancer diagnosis was evident only in women with no central obesity.^10^ Considering the impact of obesity and central obesity on insulin resistance and diabetes,^41,42,43^ we also evaluated diabetes status as a potential modifier but did not find that the dietary pattern–survival association differed by diabetes status.

### Limitations and Strengths

Our study has several limitations. Rapid case ascertainment was used to identify Black women with ovarian cancer, and comparisons of survival rates in our study with those in the SEER database suggest that our participants were representative of those who survived 10 months or more after diagnosis.^15,44^ The generalizability of our findings to women with the most fatal cancers, who die shortly after diagnosis, requires further research. In addition, we accounted for a wide array of covariates, including other lifestyle factors, but residual confounding is still possible. The median time between diagnosis and FFQ completion in our study was 5.8 months, and we acknowledge measurement errors in dietary recall as another limitation. These limitations may have contributed to the lack of an observed association for AHEI-2010 quartile 3 among women with HGSOC. Furthermore, we did not collect postdiagnostic dietary information in our study. We encourage future studies to also evaluate potential dietary changes and the association of postdiagnostic dietary quality with ovarian cancer survival.

This study also has several strengths, including the geographic diversity of the data, as they were from 11 US geographic regions. In addition to a validated and detailed FFQ, we collected extensive data, including sociodemographic, lifestyle, and clinical factors, allowing us to carefully consider confounders in the analysis. Our study adds much-needed evidence to the gap of knowledge regarding modifiable lifestyle factors associated with ovarian cancer survival among Black women. This is particularly important given that Black women have worse survival after an ovarian cancer diagnosis compared with other racial and ethnic groups.^1^

## Conclusions

This cohort study among Black women with EOC found that prediagnosis dietary quality was not associated with survival overall. However, among Black women with the most lethal form of ovarian cancer (HGSOC), moderate and high prediagnosis dietary quality were associated with better survival compared with the lowest dietary quality level. Our findings suggest that future dietary interventions should target women with the lowest dietary quality level and that even moderate adherence to dietary guidelines may be associated with improved survival after an HGSOC diagnosis.

## Notes

Supplement 1. **eTable 1.** Scoring Criteria and Distribution of Healthy Eating Index-2020 and Alternative Healthy Eating Index-2010 Among 483 Women in the African American Cancer Epidemiology Study **eTable 2.** Contribution of Each Component Score to the Association of Healthy Eating Index-2020 and Alternate Healthy Eating Index-2010 With Overall Survival Among Women With High-Grade Serous Ovarian Cancer **eTable 3.** Association of Healthy Eating Index-2020 and Alternate Healthy Eating Index-2010 with Overall Survival Among Women in the African American Cancer Epidemiology Study by Stage **eTable 4.** Association of Healthy Eating Index-2020 and Alternate Healthy Eating Index-2010 with Overall Survival Among Women in the African American Cancer Epidemiology Study by Debulking Status

Supplement 2.
Data Sharing Statement
