# Supplementary material for: Diet and Survival in Black Women With Epithelial Ovarian Cancer
Source: JAMA Netw Open. 2024 Oct 18;7(10):e2440279. doi: 10.1001/jamanetworkopen.2024.40279 (PMC11581655; doi:10.1001/jamanetworkopen.2024.40279)
Supplement: Supplement 1. — eTable 1. Scoring Criteria and Distribution of Healthy Eating Index-2020 and Alternative Healthy Eating Index-2010 Among 483 Women in the African American Cancer Epidemiology Study eTable 2. Contribution of Each Component Score to the Association of Healthy Eating Index-2020 and Alternate Healthy Eating Index-2010 With Overall Survival Among Women With High-Grade Serous Ovarian Cancer eTable 3. Association of Healthy Eating Index-2020 and Alternate Healthy Eating Index-2010 with Overall Survival Among Women in the African American Cancer Epidemiology Study by Stage eTable 4. Association of Healthy Eating Index-2020 and Alternate Healthy Eating Index-2010 with Overall Survival Among Women in the African American Cancer Epidemiology Study by Debulking Status [file jamanetwopen-e2440279-s001.pdf]

# Supplemental Online Content

Armidie TA, Bandera EV, Johnson CE, et al. Diet and survival in Black women with epithelial ovarian cancer. *JAMA Netw Open*. 2024;7(10):e2440279. doi:10.1001/jamanetworkopen.2024.40279

**eTable 1.** Scoring Criteria and Distribution of Healthy Eating Index-2020 and Alternative Healthy Eating Index-2010 Among 483 Women in the African American Cancer Epidemiology Study

**eTable 2.** Contribution of Each Component Score to the Association of Healthy Eating Index-2020 and Alternate Healthy Eating Index-2010 With Overall Survival Among Women With High-Grade Serous Ovarian Cancer

**eTable 3.** Association of Healthy Eating Index-2020 and Alternate Healthy Eating Index-2010 with Overall Survival Among Women in the African American Cancer Epidemiology Study by Stage

**eTable 4.** Association of Healthy Eating Index-2020 and Alternate Healthy Eating Index-2010 with Overall Survival Among Women in the African American Cancer Epidemiology Study by Debulking Status

This supplemental material has been provided by the authors to give readers additional information about their work.

© 2024 Armidie TA et al. *JAMA Network Open*.

**eTable 1.** Scoring Criteria and Distribution of Healthy Eating Index–2020 and Alternative Healthy Eating Index–2010 Among Women in the African American Cancer Epidemiology Study (n=483)

| HEI-2020 Components | Scoring Criteria | | | All, Mean Score (SD) | Quartiles of HEI-2010, Mean Score (SD) | | | |
|---|---|---|---|---|---|---|---|---|
| | Minimum Score (0) | Maximum Score (5 or 10) | | | Q1 | Q2 | Q3 | Q4 |
| | | | | (n=483) | (n=121) | (n=121) | (n=121) | (n=120) |
| Score ranges | | | | | ≤61.03 | 61.04-67.93 | 67.94-74.20 | ≥74.21 |
| Total Fruits | No Fruit | ≥0.8 cup equivalents/1,000 kcal | 5 | 3.70 (1.40) | 2.50 (1.35) | 3.62 (1.30) | 4.09 (1.17) | 4.60 (0.79) |
| Whole Fruits | No Whole Fruit | ≥0.4 cup equivalents/1,000 kcal | 5 | 3.77 (1.40) | 2.58 (1.29) | 3.80 (1.31) | 4.10 (1.23) | 4.59 (0.90) |
| Total Vegetables | No Vegetables | ≥1.1 cup equivalents/1,000 kcal | 5 | 3.68 (1.24) | 2.81 (1.18) | 3.50 (1.09) | 4.02 (1.10) | 4.38 (1.00) |
| Greens and Beans | No Dark Green Vegetables or Beans and Peas | ≥0.2 cup equivalents/1,000 kcal | 5 | 4.25 (1.27) | 3.64 (1.52) | 4.21 (1.29) | 4.45 (1.12) | 4.71 (0.82) |
| Whole Grains | No Whole Grains | ≥1.5 oz equivalents/1,000 kcal | 10 | 3.74 (2.59) | 2.71 (2.07) | 2.97 (2.32) | 3.95 (2.49) | 5.36 (2.61) |
| Dairy | No Dairy | ≥1.3 cup equivalents/1,000 kcal | 10 | 4.12 (2.25) | 3.15 (1.41) | 3.55 (1.89) | 4.59 (2.34) | 5.22 (2.57) |
| Total Protein Foods | No Protein Foods | ≥2.5 oz equivalents/1,000 kcal | 5 | 4.65 (0.66) | 4.54 (0.70) | 4.71 (0.64) | 4.68 (0.65) | 4.67 (0.64) |
| Seafood and Plant Proteins | No Seafood and Plant Proteins | ≥0.8 oz equivalents/1,000 kcal | 5 | 4.11 (1.23) | 3.18 (1.34) | 4.10 (1.25) | 4.47 (0.93) | 4.69 (0.72) |
| Fatty Acids | (PUFAs+MUFAs)/SFAs ≤1.2 | (PUFAs+MUFAs)/SFAs ≥2.5 | 10 | 6.91 (2.32) | 5.92 (2.13) | 6.77 (2.22) | 6.84 (2.21) | 8.12 (2.21) |
| Refined Grains | ≥4.3 oz equivalents/1,000 kcal | ≤1.8 oz equivalents/1,000 kcal | 10 | 8.60 (1.93) | 7.56 (2.44) | 8.25 (2.05) | 9.08 (1.23) | 9.54 (1.01) |
| Sodium | ≥2.0 g/1,000 kcal | ≤1.1 g/1,000 kcal | 10 | 4.01 (2.81) | 4.11 (2.94) | 3.96 (2.98) | 3.71 (2.65) | 4.25 (2.67) |
| Added Sugars | ≥26% of energy | ≤6.5% of energy | 10 | 6.08 (3.08) | 4.73 (3.45) | 5.78 (3.18) | 6.44 (2.61) | 7.39 (2.38) |
| Saturated Fats | ≥16% of energy | ≤8% of energy | 10 | 6.12 (2.45) | 5.14 (2.56) | 5.82 (2.39) | 6.22 (2.34) | 7.29 (2.03) |
| **Total** | **0** | **100** | | **67.87 (9.49)** | 55.73 (4.28) | 64.60 (2.10) | 71.23 (1.89) | 80.01 (4.13) |

| AHEI-2010 Components | Scoring Criteria | | All, Mean Score (SD) | Quartiles of AHEI-2010, Mean Score (SD) | | | |
|---|---|---|---|---|---|---|---|
| | Minimum Score (0) | Maximum Score (10) | | Q1 | Q2 | Q3 | Q4 |
| | | | (n=483) | (n=121) | (n=121) | (n=121) | (n=120) |
| Score ranges | | | | ≤45.33 | 45.34-52.47 | 52.48-59.68 | ≥59.69 |
| Vegetables | 0 servings/day | ≥5 servings/day | 4.71 (2.85) | 3.18 (1.93) | 3.89 (2.45) | 4.99 (2.76) | 6.78 (2.85) |
| Fruit | 0 servings/day | ≥4 servings/day | 3.55 (2.73) | 2.01 (1.56) | 3.07 (2.15) | 3.71 (2.70) | 5.45 (3.06) |
| Whole grains | 0 g/day | ≥75 g/day | 2.08 (2.31) | 1.50 (1.48) | 1.60 (1.58) | 2.07 (2.27) | 3.16 (3.16) |
| Nuts and legumes | 0 servings/day | ≥1 servings/day | 4.21 (3.38) | 2.19 (1.95) | 3.39 (2.98) | 4.48 (3.37) | 6.80 (3.20) |
| Red/processed meat | ≥1.5 servings/day | 0 servings/day | 5.50 (2.98) | 4.14 (2.82) | 5.45 (2.82) | 5.68 (2.95) | 6.73 (2.79) |
| Sugar-sweetened beverages and fruit juice | ≥1 servings/day | 0 servings/day | 2.78 (3.70) | 0.60 (1.86) | 1.92 (3.12) | 3.35 (3.78) | 5.28 (3.93) |

© 2024 Armidie TA et al. *JAMA Network Open.*

| | | | | | | | |
|---|---|---|---|---|---|---|---|
| Trans Fat | ≥4% of energy | ≤0.5% of energy | 8.02 (1.15) | 7.60 (1.26) | 7.79 (1.11) | 8.15 (0.91) | 8.55 (1.07) |
| Long-chain (n-3) fats (EPA + DHA) | 0 mg/day | ≥250 mg/day | 3.63 (2.71) | 2.67 (1.91) | 3.31 (2.49) | 4.10 (2.97) | 4.46 (2.98) |
| PUFA | ≤2% of energy | ≥10% of energy | 8.43 (1.39) | 7.76 (1.52) | 8.15 (1.44) | 8.67 (1.16) | 9.13 (1.00) |
| Sodium | Highest decile | Lowest decile | 6.06 (3.21) | 5.85 (2.95) | 6.35 (3.34) | 6.18 (3.32) | 5.85 (3.20) |
| Alcohol | ≥2.5 drinks/day | 0.5–1.5 drinks/day | 4.14 (1.90) | 3.35 (1.50) | 4.11 (1.67) | 4.44 (2.08) | 4.67 (2.02) |
| **Total** | **0** | **110** | **53.12 (10.23)** | 40.85 (3.73) | 49.04 (2.10) | 55.81 (2.05) | 66.87 (5.78) |

HEI-2020, healthy eating index; AHEI-2010, alternative healthy eating index

© 2024 Armidie TA et al. *JAMA Network Open.*

**eTable 2.** The Influence of Each Component Score on the Association of the Healthy Eating Index–2020 and Alternate Healthy Eating Index–2010 with Overall Survival Among Women with High-Grade Serous Ovarian Cancer

| Components | Scoring Criteria | | Model 1[a,c] | Model 2[b,c] |
|---|---|---|---|---|
| | Minimum Score (0) | Maximum Score (10 or 5) | HR (95% CI) | HR (95% CI) |
| **HEI-2020** | | | | |
| Total Fruits | No Fruit | ≥0.8 cup equivalents/1,000 kcal | 1.05 (0.94, 1.16) | 0.84 (0.72, 0.99) |
| Whole Fruits | No Whole Fruit | ≥0.4 cup equivalents/1,000 kcal | 0.97 (0.87, 1.08) | 0.86 (0.73, 1.00) |
| Total Vegetables | No Vegetables | ≥1.1 cup equivalents/1,000 kcal | 0.93 (0.83, 1.05) | 0.86 (0.74, 1.01) |
| Greens and Beans | No Dark Green Vegetables or Beans and Peas | ≥0.2 cup equivalents/1,000 kcal | 0.94 (0.85, 1.05) | 0.87 (0.74, 1.01) |
| Whole Grains | No Whole Grains | ≥1.5 oz equivalents/1,000 kcal | 0.98 (0.94, 1.03) | 0.86 (0.74, 1.00) |
| Dairy | No Dairy | ≥1.3 cup equivalents/1,000 kcal | 1.03 (0.97, 1.10) | 0.84 (0.72, 0.97) |
| Total Protein Foods | No Protein Foods | ≥2.5 oz equivalents/1,000 kcal | 1.23 (0.98, 1.56) | 0.85 (0.74, 0.99) |
| Seafood and Plant Proteins | No Seafood and Plant Proteins | ≥0.8 oz equivalents/1,000 kcal | 0.94 (0.84, 1.04) | 0.87 (0.75, 1.01) |
| Fatty Acids | (PUFAs+MUFAs)/SFAs ≤1.2 | (PUFAs+MUFAs)/SFAs ≥2.5 | 0.95 (0.90, 1.01) | 0.88 (0.75, 1.03) |
| Refined Grains | ≥4.3 oz equivalents/1,000 kcal | ≤1.8 oz equivalents/1,000 kcal | 0.99 (0.92, 1.06) | 0.85 (0.73, 1.00) |
| Sodium | ≥2.0 g/1,000 kcal | ≤1.1 g/1,000 kcal | 0.96 (0.91, 1.01) | 0.89 (0.78, 1.02) |
| Added Sugars | ≥26% of energy | ≤6.5% of energy | 1.01 (0.97, 1.05) | 0.84 (0.73, 0.98) |
| Saturated Fats | ≥16% of energy | ≤8% of energy | 0.95 (0.90, 1.00) | 0.88 (0.76, 1.03) |
| **AHEI 2010** | | | | |
| Vegetables | 0 servings/day | ≥5 servings/day | 1.02 (0.97, 1.08) | 0.85 (0.73,0.99) |
| Fruit | 0 servings/day | ≥4 servings/day | 1.01 (0.96, 1.06) | 0.86 (0.74,1.00) |
| Whole Grains | 0 servings/day | ≥75 g/day | 0.98 (0.93, 1.04) | 0.89 (0.77, 1.02) |
| Nuts and Legumes | 0 servings/day | ≥1 servings/day | 1.02 (0.98, 1.06) | 0.85 (0.72, 0.99) |
| Red/processed Meat | ≥1.5 servings/day | 0 servings/day | 0.94 (0.90, 0.98) | 0.93 (0.81, 1.07) |
| Sugar-sweetened Beverages and Fruit Juice | ≥1 servings/day | 0 servings/day | 0.99 (0.95, 1.03) | 0.88 (0.75, 1.02) |
| Trans Fat | ≥4% of energy | ≤0.5% of energy | 1.01 (0.90, 1.13) | 0.88 (0.76, 1.01) |
| Long-chain (n-3) Fats (EPA + DHA) | 0 mg/day | ≥250 mg/day | 1.01 (0.96, 1.06) | 0.87 (0.75, 1.00) |
| PUFA | ≤2% of energy | ≥10% of energy | 0.99 (0.90, 1.10) | 0.88 (0.76, 1.02) |
| Sodium | Highest decile mg/day | Lowest decile mg/day | 0.95 (0.91, 1.00) | 0.93 (0.82, 1.05) |
| Alcohol | ≥2.5 drinks/day | 0.5–1.5 drinks/day | 0.98 (0.91, 1.05) | 0.89 (0.77, 1.02) |

a. Estimated association between a 1-point increase in each dietary component score and survival, adjusted for the overall score without the component under study.
b. Estimated association between a 10-point increase in dietary pattern score and survival, after removing the dietary component under study.
c. Both Cox models also adjusted for age, education, household income, physical activity, smoking status, and study site.

© 2024 Armidie TA et al. *JAMA Network Open.*

**eTable 3.** Association of the Healthy Eating Index–2020 and Alternate Healthy Eating Index–2010 with Overall Survival Among Women in the African American Cancer Epidemiology by Stage[a,b,c]

| | HEI-2020 | | | | AHEI-2010 | | | |
|---|---|---|---|---|---|---|---|---|
| | Stage I & II[d] | | Stage III & IV | | Stage I & II[d] | | Stage III & IV | |
| **Dietary Pattern Score** | **Deaths/ Participants No.** | **HR (95%CI)** | **Deaths/ Participants No.** | **HR (95%CI)** | **Deaths/ Participants No.** | **HR (95%CI)** | **Deaths/ Participants No.** | **HR (95%CI)** |
| Quartile [Q] 1 low adherence | 7/35 | 1 (reference) | 70/81 | 1 (reference) | 9/32 | 1 (reference) | 70/84 | 1 (reference) |
| Q2 | 11/35 | 1.52 (0.55, 4.18) | 59/79 | 0.70 (0.49, 0.99) | 10/36 | 0.91 (0.35, 2.39) | 58/75 | 0.79 (0.55, 1.13) |
| Q3 | 15/39 | 1.52 (0.59, 3.91) | 60/74 | 0.90 (0.63, 1.28) | 15/37 | 1.23 (0.51, 2.95) | 66/80 | 1.10 (0.78, 1.56) |
| Q4 high adherence | 14/41 | 1.23 (0.46, 3.26) | 56/71 | 0.81 (0.56, 1.17) | 13/45 | 0.78 (0.31, 1.96) | 51/66 | 0.96 (0.65, 1.41) |
| *p*-trend | | 0.80 | | 0.44 | | 0.64 | | 0.79 |
| Per 10-point increase | | 0.95 (0.70, 1.30) | | 0.96 (0.83, 1.11) | | 1.00 (0.74, 1.35) | | 0.96 (0.84, 1.11) |

Abbreviations: HEI-2020, healthy eating index; AHEI-2010, alternative healthy eating index.

[a]HEI-2020 score range: 0-100; AHEI-2010 score range: 0-110.

[b]Cox proportional hazard model adjusted for age, education, household income, physical activity, smoking status, and study site.

[c]HEI-2020: *p*-interaction=0.22; AHEI-2010: *p*-interaction=0.78

[d]Results should be interpreted with caution due to the small sample size in the early stages

© 2024 Armidie TA et al. *JAMA Network Open.*

**eTable 4.** Association of the Healthy Eating Index–2020 and Alternate Healthy Eating Index–2010 with Overall Survival Among Women in the African American Cancer Epidemiology by Debulking Status[a,b,c,d]

| | HEI-2020 | | | | AHEI-2010 | | | |
|---|---|---|---|---|---|---|---|---|
| | **Optimal Debulking** | | **Suboptimal Debulking** | | **Optimal Debulking** | | **Suboptimal Debulking** | |
| **Dietary Pattern Score** | **Deaths/ Participants No.** | **HR (95%CI)** | **Deaths/ Participants No.** | **HR (95%CI)** | **Deaths/ Participants No.** | **HR (95%CI)** | **Deaths/ Participants No.** | **HR (95%CI)** |
| Quartile [Q] 1 low adherence | 43/80 | 1 (reference) | 37/41 | 1 (reference) | 44/80 | 1 (reference) | 39/41 | 1 (reference) |
| Q2 | 47/88 | 0.83 (0.54, 1.27) | 30/33 | 0.84 (0.50, 1.43) | 44/87 | 0.88 (0.57, 1.35) | 30/34 | 0.61 (0.37, 1.00) |
| Q3 | 39/78 | 0.75 (0.48, 1.16) | 39/43 | 1.11 (0.69, 1.80) | 46/80 | 1.16 (0.75, 1.77) | 37/41 | 0.83 (0.51, 1.34) |
| Q4 high adherence | 50/94 | 0.83 (0.54, 1.27) | 25/26 | 1.00 (0.56, 1.76) | 45/93 | 0.88 (0.56, 1.37) | 25/27 | 0.71 (0.39, 1.28) |
| *p*-trend | | 0.38 | | 0.73 | | 0.82 | | 0.40 |
| Per 10-point increase | | 0.93 (0.79, 1.09) | | 1.03 (0.82, 1.28) | | 0.97 (0.83, 1.12) | | 0.93 (0.75, 1.15) |

Abbreviations: HEI-2020, healthy eating index; AHEI-2010, alternative healthy eating index.

[a]HEI-2020 score range: 0-100; AHEI-2010 score range: 0-110.

[b]Cox proportional hazard model, adjusted for age, education, household income, physical activity, smoking status, and study site.

[c]HEI-2020: *p*-interaction=0.77; AHEI-2010: *p*-interaction=0.55

© 2024 Armidie TA et al. *JAMA Network Open.*
