# Supplementary material for: Diet and Survival in Black Women With Epithelial Ovarian Cancer
Source: JAMA Netw Open. 2024 Oct 18;7(10):e2440279. doi: 10.1001/jamanetworkopen.2024.40279 (PMC11581655; doi:10.1001/jamanetworkopen.2024.40279)
Supplement: Supplement 2. — Data Sharing Statement [file jamanetwopen-e2440279-s002.pdf]

Data Sharing Statement

Armidie. Diet and Survival in Black Women With Epithelial Ovarian Cancer. *JAMA Netw Open*. Published online October 18. 2024. doi:10.1001/jamanetworkopen.2024.40279

Data
Data available: Yes
Data types: Deidentified participant data, Data dictionary
How to access data: De-identified data and data dictionary for this current study is available upon reasonable request, in accordance with NIH data sharing policy. Inquiries can be directed to:aaces@emory.edu
When available: With publication

Supporting Documents
Document types: None

Additional Information
Who can access the data: Researchers whose proposed use of the data has been approved
Types of analyses: For research purposes.
Mechanisms of data availability: After approval of a proposal, and with a signed data access agreement.
